# Supplementary material for: A functional map of CDK-drug interactions at single amino acid resolution
Source: bioRxiv. 2025 Nov 3:2025.11.02.685764. Preprint. [Version 1] doi: 10.1101/2025.11.02.685764 (PMC12637707; doi:10.1101/2025.11.02.685764)
Supplement: Supplement 7 [file NIHPP2025.11.02.685764v1-supplement-7.pdf]

bioRxiv preprint doi: https://doi.org/10.1101/2025.11.02.685764; this version posted November 3, 2025. The copyright holder for this preprint (which was not certified by peer review) is the author/funder, who has granted bioRxiv a license to display the preprint in perpetuity. It is made available under aCC-BY 4.0 International license.

Supplementary Tables

- **Supplementary Table 1**: CDK base editing tiling library
- **Supplementary Table 2**: Editing information for ABE screens
- **Supplementary Table 3**: Editing information for CBE screens
- **Supplementary Table 4:** LFC/FDR information for ABE screens
- **Supplementary Table 5**: LFC/FDR information for CBE screens
- **Supplementary Table 6:** Sequences of individually tested gRNAs

bioRxiv preprint doi: https://doi.org/10.1101/2025.11.02.685764; this version posted November 3, 2025. The copyright holder for this preprint (which was not certified by peer review) is the author/funder, who has granted bioRxiv a license to display the preprint in perpetuity. It is made available under aCC-BY 4.0 International license.

a

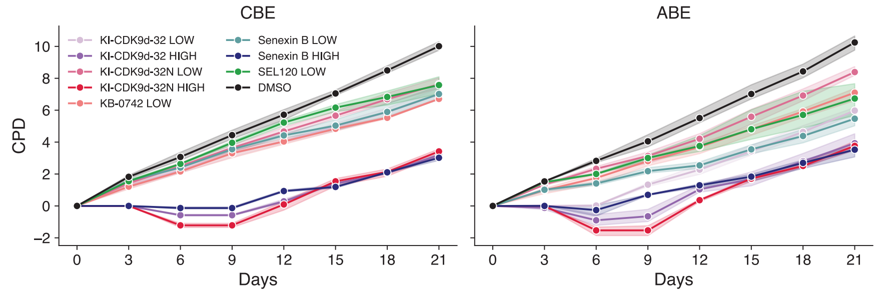

Dose (µM)

	CBE							ABE						
KI-CDK9d-32 LOW:	0.1	0.1	0.1	0.1	0.1	0.1	0.1	0.1	0.1	0.1	0.1	0.1	0.1	0.1
KI-CDK9d-32 HIGH:	1.0	1.0	0	0	0	0.2	0.2	1.0	1.0	0	0	0.2	0.2	0.2
KI-CDK9d-32N LOW:	1.25	1.25	1.25	1.25	1.25	1.25	1.25	1.25	1.25	1.25	1.25	1.25	1.25	1.25
KI-CDK9d-32N HIGH:	5.0	5.0	0	0	0	2.0	2.0	5.0	5.0	0	0	0	2.0	2.0
KB-0742 LOW:	1.5	1.5	1.5	1.5	1.5	1.5	1.5	1.5	1.5	1.5	1.5	1.5	1.5	1.5
Senexin B LOW:	2.0	2.0	2.0	2.0	2.0	2.0	2.0	2.0	2.0	2.0	2.0	2.0	2.0	2.0
Senexin B HIGH:	15.0	15.0	0	0	4.0	0	4.0	15.0	15.0	0	4.0	4.0	4.0	4.0
SEL120 LOW:	4.0	4.0	4.0	4.0	4.0	4.0	4.0	4.0	4.0	4.0	4.0	4.0	4.0	4.0

b

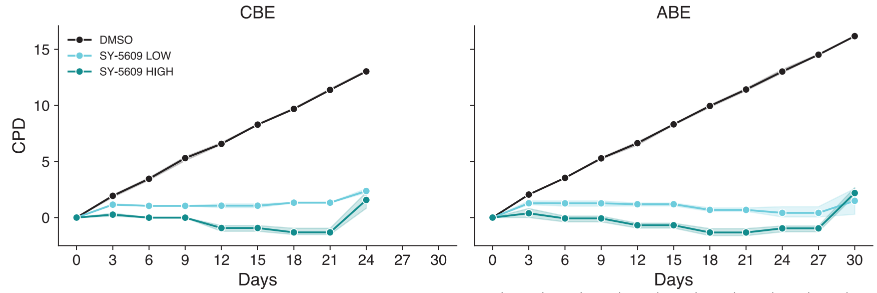

Dose (nM)

	CBE								ABE									
SY-5609 LOW:	10	100	100	100	100	100	10	10	10	100	100	100	100	100	10	10	0	0
SY-5609 HIGH:	100	1,000	1,000	1,000	0	0	0	0	100	1,000	1,000	1,000	0	0	0	0	0	0

c

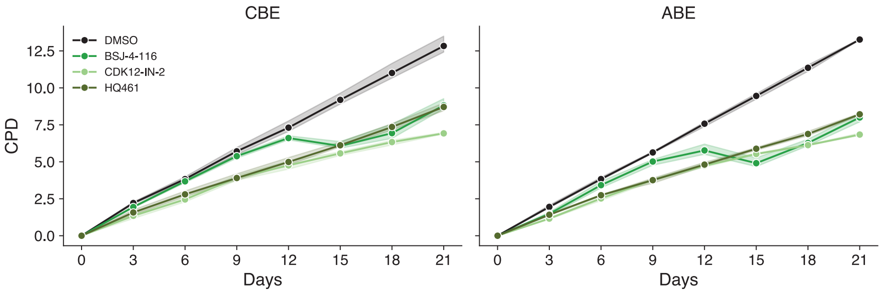

Dose (µM)

	CBE							ABE						
BSJ-4-116:	0.13	0.13	0.13	0.26	0.52	0	0.13	0.13	0.13	0.13	0.26	0.52	0	0.13
CDK12-IN-2:	2.25	2.25	2.25	3.0	3.0	3.0	3.0	2.25	2.25	2.25	3.0	3.0	3.0	3.0
HQ461:	1.7	1.7	1.7	1.7	1.7	1.7	1.7	1.7	1.7	1.7	1.7	1.7	1.7	1.7

d

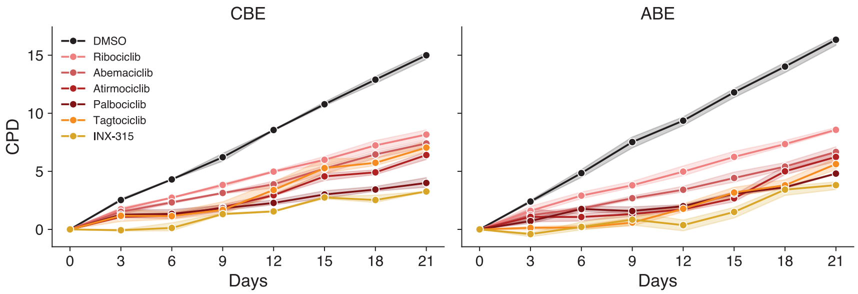

Dose (µM)

	CBE							ABE						
Ribociclib:	10.0	10.0	10.0	10.0	10.0	10.0	10.0	10.0	10.0	10.0	10.0	10.0	10.0	10.0
Abemaciclib:	4.0	4.0	4.0	4.0	2.0	2.0	2.0	4.0	4.0	4.0	4.0	2.0	2.0	2.0
Atirmociclib:	5.0	5.0	0	2.5	2.5	5.0	0	5.0	5.0	0	2.5	2.5	5.0	0
Palbociclib:	8.0	8.0	0	6.0	0	6.0	0	8.0	8.0	0	6.0	0	6.0	0
Tagtociclib:	2.0	2.0	0	1.2	1.5	2.0	0	2.0	2.0	0	1.2	1.5	2.0	0
INX-315:	5.0	0	0	2.5	0	2.5	1.0	5.0	0	0	2.5	0	2.5	1.0

bioRxiv preprint doi: https://doi.org/10.1101/2025.11.02.685764; this version posted November 3, 2025. The copyright holder for this preprint (which was not certified by peer review) is the author/funder, who has granted bioRxiv a license to display the preprint in perpetuity. It is made available under aCC-BY 4.0 International license.

Supplementary Figure 1. Cumulative population doublings in each screen. Cumulative population doublings (CPD), defined as the sum of log_2(cell count/cells plated) for **(a)** the subpool 1 screen, **(b)** the re-screen of subpool 1 in the presence of SY-5609, **(c)** the subpool 2 screen, and **(d)** the subpool 3 screen. Points represent the average CPD value across three replicates, with the shaded region indicating the 95% confidence interval of the mean value. Drug doses at each timepoint, which had to be adjusted for certain conditions based on cell count and morphology, are indicated below each plot.

bioRxiv preprint doi: https://doi.org/10.1101/2025.11.02.685764; this version posted November 3, 2025. The copyright holder for this preprint (which was not certified by peer review) is the author/funder, who has granted bioRxiv a license to display the preprint in perpetuity. It is made available under aCC-BY 4.0 International license.

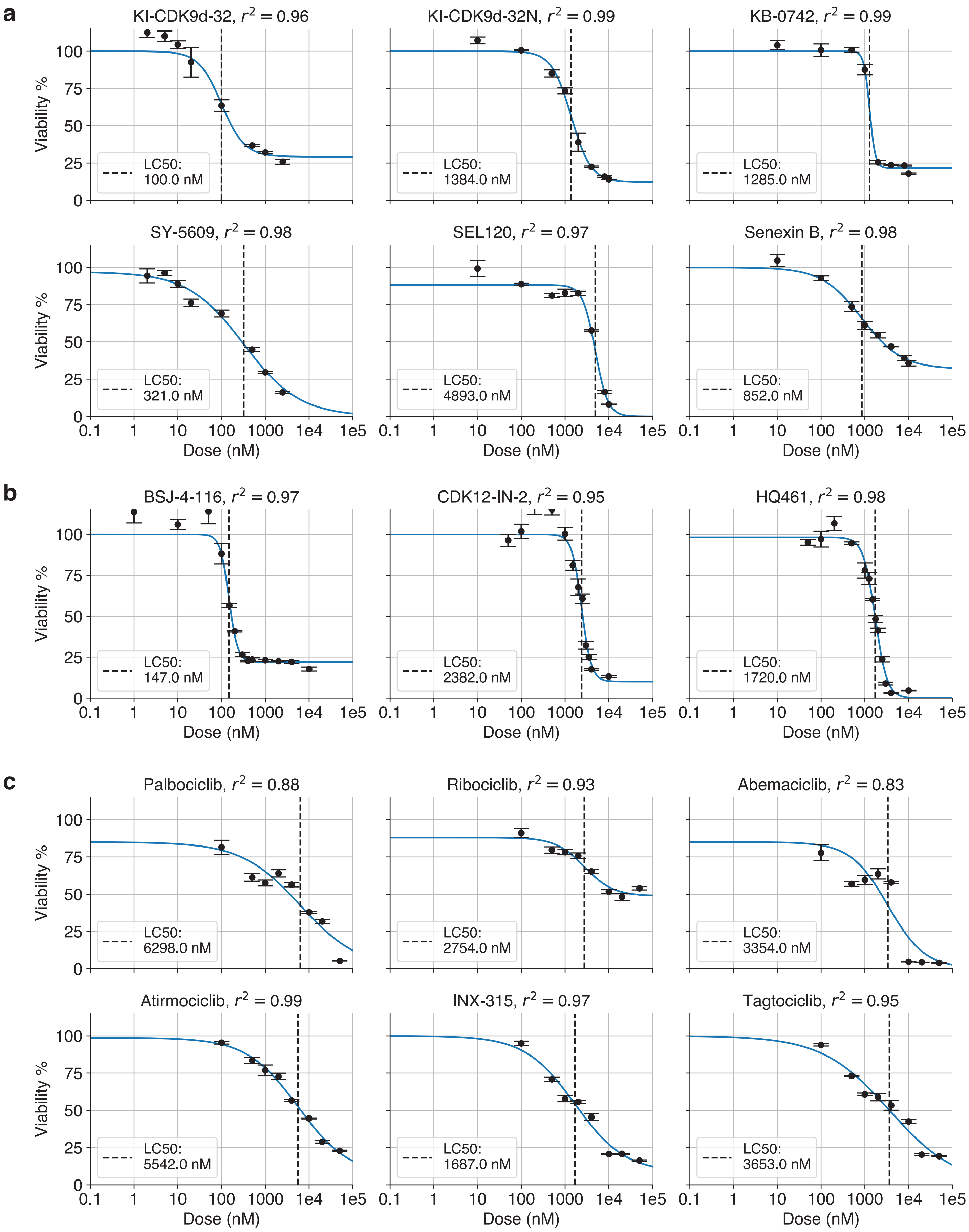

bioRxiv preprint doi: https://doi.org/10.1101/2025.11.02.685764; this version posted November 3, 2025. The copyright holder for this preprint (which was not certified by peer review) is the author/funder, who has granted bioRxiv a license to display the preprint in perpetuity. It is made available under aCC-BY 4.0 International license.

Supplementary Figure 2. Dose-response curves for each drug. Dose-response curves for drugs used in **(a)** subpool 1, **(b)** subpool 2, and **(c)** subpool 3. Error bars at each point indicate standard deviation of three technical replicates. Curves fit with four parameter log-logistic function, with LC50 calculated as the concentration where half of the maximal cell death occurs.

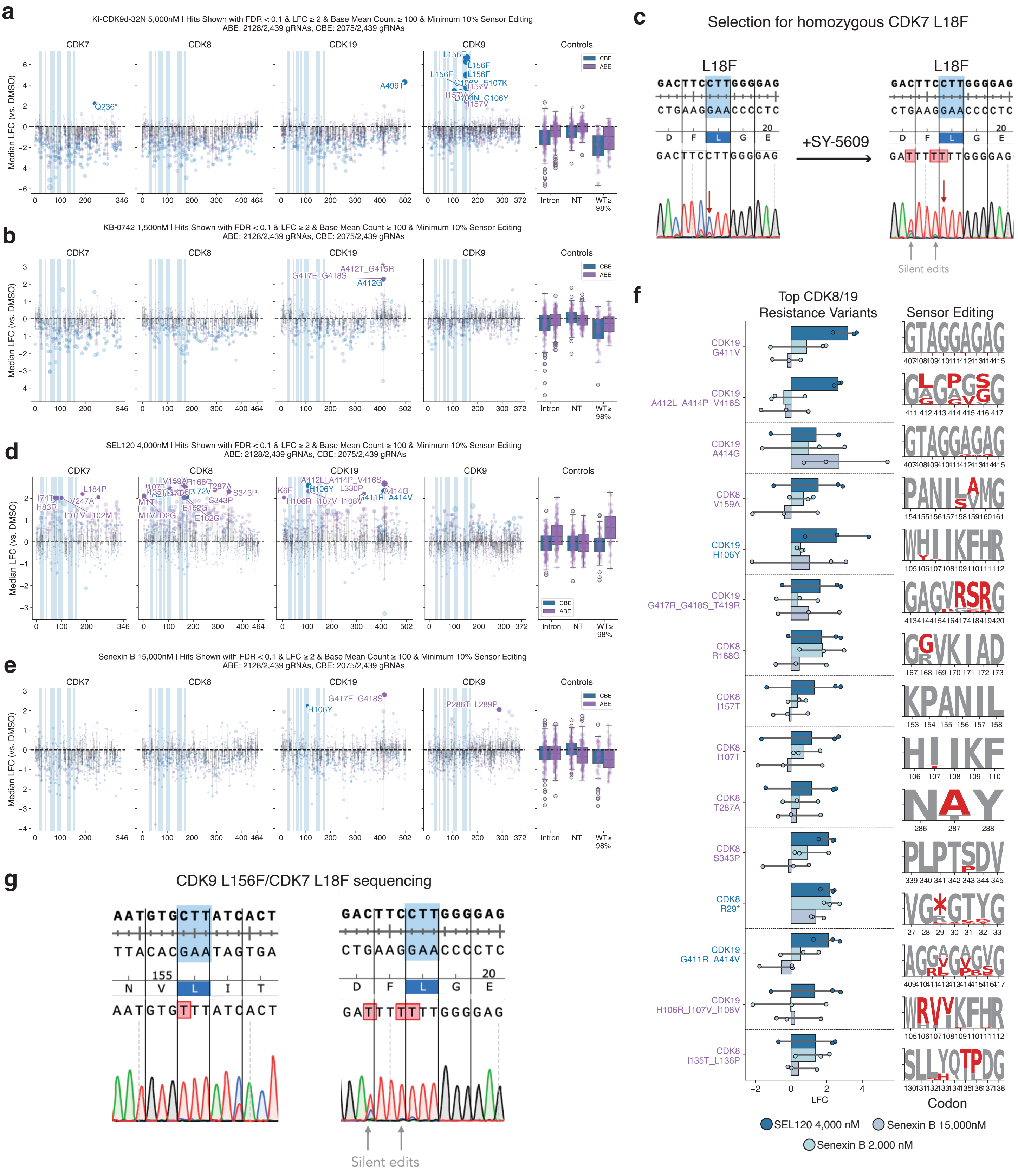

bioRxiv preprint doi: https://doi.org/10.1101/2025.11.02.685764; this version posted November 3, 2025. The copyright holder for this preprint (which was not certified by peer review) is the author/funder, who has granted bioRxiv a license to display the preprint in perpetuity. It is made available under aCC-BY 4.0 International license.

Supplementary Figure 3. Additional information about subpool 1 and iterative screens. a) Scatterplot of median gRNA enrichment ($\log_2$ fold-change) in the presence of 5,000 nM KI-CDK9d-32N relative to DMSO-treated control. Each dot represents a gRNA, filtered to exclude gRNAs below 10% sensor editing and with a base mean count ≤ 100. Hits labelled with FDR < 0.1 & LFC ≥ 2. ABE screen results shown in purple; CBE in blue. Regions shaded blue indicate ATP binding site residues. **b)** Same as (a), but for cells treated with KB-0742 (1,500 nM). **c)** Sanger sequencing of cells transduced with CDK7 L18F gRNA before and after selection with SY-5609. **d)** Same as (a), but for cells treated with SEL120 (4,000 nM). **e)** Same as (a), but for cells treated with Senexin B (15,000 nM). **f)** Top resistance variants in CDK8/19, as ranked by $\log_2$ fold-change in SEL120 4,000 nM treatment condition. **g)** Sanger sequencing of CDK9 L156F/CDK7 L18F cells.

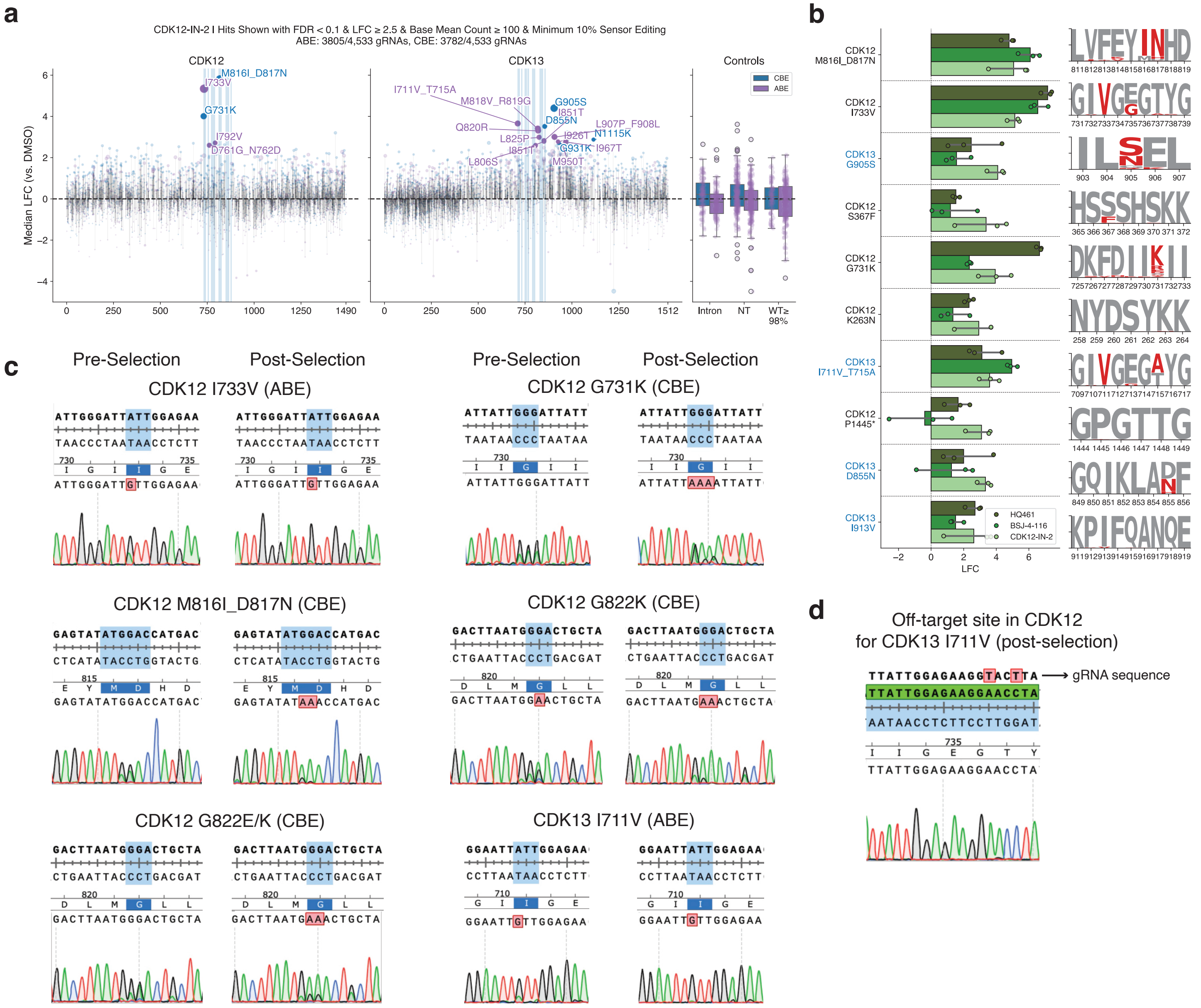

Supplementary Figure 4. Identification and validation of resistance variants in CDK12/13. a) Scatterplot of median gRNA enrichment ($\log_2$ fold-change) in the presence of CDK12-IN-2 relative to DMSO-treated control. Each dot represents a gRNA, filtered to exclude gRNAs below 10% sensor editing and with a base mean count ≤ 100. Hits labelled with FDR < 0.1 & LFC ≥ 2. ABE screen results shown in purple; CBE in blue.

bioRxiv preprint doi: https://doi.org/10.1101/2025.11.02.685764; this version posted November 3, 2025. The copyright holder for this preprint (which was not certified by peer review) is the author/funder, who has granted bioRxiv a license to display the preprint in perpetuity. It is made available under aCC-BY 4.0 International license.

Regions shaded blue indicate ATP binding site residues. **b)** Top resistance variants in CDK12/13, as ranked by $\log_2$ fold-change in CDK12-IN-2 treatment condition. **c)** Sanger sequencing of arrayed validation of CDK12/13 mutations before and after selection with HQ461. **d)** Sanger sequencing of CDK12 off-target site for cells transduced with CDK13 I711V gRNA and selected with HQ461, indicating no off-target editing.

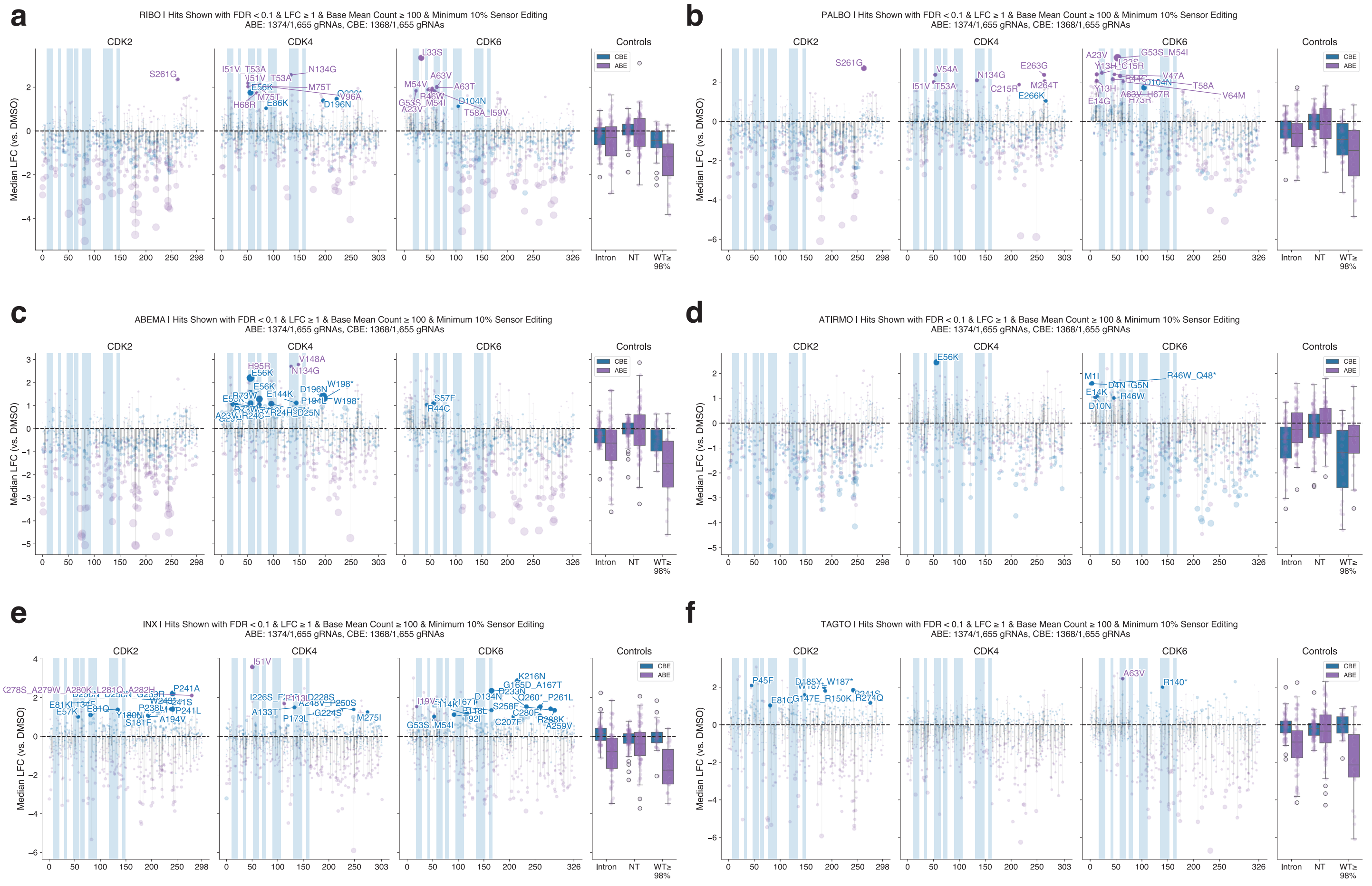

Supplementary Figure 5. Identification of resistance variants in CDK2/4/6. Scatterplots of median gRNA enrichment ($\log_2$ fold-change) in the presence of **a)** Ribociclib, **b)** Palbociclib, **c)** Abemaciclib, **d)** Atirmociclib, **e)** INX-315, or **f)** Tagtociclib relative to DMSO-treated control. Each dot represents a gRNA, filtered to exclude gRNAs below 10% sensor editing and with a base mean count ≤ 100. Hits labelled with FDR < 0.1 & LFC ≥ 1. ABE screen results shown in purple; CBE in blue. Regions shaded blue indicate ATP binding site residues.
